# Drug Repurposing: *In vitro* and *in vivo* Antimicrobial and Antibiofilm Effects of Bithionol Against *Enterococcus faecalis* and *Enterococcus faecium*

**DOI:** 10.3389/fmicb.2021.579806

**Published:** 2021-05-06

**Authors:** Pengfei She, Yangxia Wang, Yingjia Li, Linying Zhou, Shijia Li, Xianghai Zeng, Yaqian Liu, Lanlan Xu, Yong Wu

**Affiliations:** ^1^Department of Laboratory Medicine, Third Xiangya Hospital, Central South University, Changsha, China; ^2^Department of Laboratory Medicine, The First Affiliated Hospital of Zhengzhou University, Zhengzhou, China

**Keywords:** drug repurposing, bithionol, antimicrobial, Enterococci, bacterial cell membrane

## Abstract

Widespread antibiotic resistance has been reported in enterococcal pathogens that cause life-threatening infections. Enterococci species rapidly acquire resistance and the pace of new antibiotic development is slow. Drug repurposing is a promising approach in solving this problem. Bithionol (BT) is a clinically approved anthelminthic drug. In this study, we found that BT showed significant antimicrobial and antibiofilm effects against *Enterococcus faecalis* and vancomycin-resistant *Entercococcus faecium in vitro*, in a dose-dependent manner, by disrupting the integrity of the bacterial cell membranes. Moreover, BT effectively reduced the bacterial load in mouse organs when combined with conventional antibiotics in a peritonitis infection model. Thus, BT has shown potential as a therapeutic agent against *E. faecalis*- and vancomycin-resistant *E. faecium*-related infections.

## Introduction

Enterococci are Gram-positive, anaerobic bacteria that are associated with many life-threatening infections, such as bacteremia, endocarditis, and abscesses. Enterococci have developed antibiotic resistance to vancomycin (VAN), linezolid, and daptomycin (DAP) between 1980 and 2010. However, the development of antimicrobial agents is time consuming, while the emergence of antibiotic resistance in enterococci is rapid (Mercuro et al., 2018).

Enterococci adhere to the surface of medical devices or human tissues to form biofilms (Ch’ng et al., 2019). *Enterococcus faecalis* and *Enterococcus faecium* are the two major pathogens belonging to this family. However, *E. faecalis* has a greater ability to form biofilms than *E. faecium*. The worldwide prevalence of *E. faecalis* biofilm associated infections is reported to range from 57.2 to 100%, with 93% reported in the United States of America (Zheng et al., 2017). Catheter-associated urinary tract infections, surgical wound infections, persistent root canal infections, and infective endocarditis are major biofilm-related diseases caused by *E. faecalis* (Ch’ng et al., 2019). Biofilms show extremely high protection against host defenses, extremely high antimicrobial resistance, and enhanced virulence compared to their planktonic counterparts, owing to physical barriers (such as biofilm matrix containing extracellular DNA, *epa*-coding polysaccharides, and Esp/GelE surface proteins) and formation of persister cells (Mohamed and Huang, 2007; Flemming and Wingender, 2010; Paganelli et al., 2012).

Bithionol (BT), a clinically approved anthelminthic drug, was found to be active against methicillin-resistant *Staphylococcus aureus* (MRSA) and its persister cells *in vitro* and *in vivo*, by disrupting bacterial cell membrane lipid bilayers (Kim et al., 2019). The toxicity, pharmacokinetics, and safety profiles of BT are well established (Keiser and Utzinger, 2007), and it shows great promise as a potential antibiotic for clinical use.

However, to the best of our knowledge, no systematic studies have been conducted on the *in vitro* and *in vivo* antimicrobial effects of BT against *E. faecalis* and VAN-resistant *E. faecium*. In this study, we aimed to describe the antimicrobial and antibiofilm effects of BT against enterococci *in vitro*, and in a mouse peritonitis infection model *in vivo*. Moreover, using previously reported methods (Kim et al., 2019), we found that the major antimicrobial target of BT against enterococci is the cell membrane lipid bilayer.

## Materials and Methods

### Strains, Culture Conditions and Chemicals

*Enterococcus faecalis* ATCC 29212 was provided by Juncai Luo (Tiandiren Biotech, Changsha, China). Clinical isolates of *E. faecalis* and *E. faecium* were obtained from urine, blood, and pleural effusion samples collected from patients at the Third Xiangya Hospital of Central South University, Changsha, China and identified using colony morphology and matrix-assisted laser desorption/ionization time-of-flight mass spectrometry (MLDI-TOF-MS, Bruker, Germany). The antimicrobial susceptibility of the clinical isolates was analyzed using bioMerieux ATB (France). The resistant pattern and other details are described in Supplementary Table S1 (She et al., 2019). The clinical isolates were stored at −80°C in a whole milk medium. *E. faecalis* and *E. faecium* were grown in brain heart infusion (BHI) broth medium (Solarbio, Shanghai, China) at 37°C. Cation-adjusted Mueller-Hinton II (MH) broth (BD/Difco, United States) was used for antimicrobial susceptibility testing. BT and antibiotics [tobramycin (TOB), gentamycin (GEN), amikacin (AMK), clindamycin hydrochloride (CLI), ceftriaxone sodium (CRO), teicoplanin (TEC), DAP, ampicillin (AMP), and VAN] were purchased from MedChemEpress (NJ, United States). BT was dissolved in dimethyl sulfoxide (DMSO), and 0.05% DMSO was used as a vehicle control throughout the experiments. The inoculum was quantified by adjusting the bacterial suspension to 0.5 McFarland (∼1 × 10^8^ CFU/mL) and then diluted to the final concentration required for each assay.

### Antimicrobial Susceptibility Test

Antimicrobial susceptibility tests were performed according to the recommendations of the Clinical and Laboratory Standards Institute 2020 (CLSI) guidelines (CLSI-M100, Performance Standards for Antimicrobial Susceptibility Testing, 30th edition). The bacterial cells were cultured overnight at 37°C with shaking at 180 rpm, followed by adjustment to 0.5 McFarland and further diluted to ∼2 × 10^5^ CFU/mL in MH broth. Equal aliquots of bacterial suspension and two-fold diluted antimicrobial agents (1,024–0.0313 μg/mL; DAP was added with 50 μg/mL of Ca^2+^) were mixed in a 96-well plate to obtain a final concentration of ∼1 × 10^5^ CFU/mL. After incubation at 37°C for 16–24 h, the minimal antimicrobial concentration necessary to inhibit the growth of the test bacteria was considered the minimum inhibitory concentration (MIC). Then, 20 μL of suspension from 1 × MIC to 64 × MIC was spread onto 5% sheep blood agar (Autobio, Zhengzhou, China) for overnight culture, and the minimum bactericidal concentration (MBC) was identified as the lowest concentration of an antimicrobial agent that killed 99.9% of the test bacteria.

### Time-Kill Assay

Overnight cultured *E. faecalis* and *E. faecium* were sub-cultured with BHI broth to the exponential phase. The bacteria were then diluted with BHI broth containing two-fold diluted antimicrobial agents to obtain a final concentration of ∼1 × 10^6^ CFU/mL in 50 mL centrifuge tubes. BHI containing 0.05% DMSO was used as a control. The tubes were incubated at 37°C with shaking at 180 rpm, and 10 μL of the bacterial suspension was removed from the tubes to perform bacterial live cell counting at the indicated time points over a period of 24 h (Ma et al., 2019).

### Dose-Dependent Inhibition

Fifty microliters of BHI containing 0–10 μg/mL of BT were added to a microplate. Enterococcal cultures in the exponential phase were diluted with BHI, and 50 μL of each bacterial suspension was diluted and added to these wells to obtain a final concentration of ∼1 × 10^6^ CFU/mL. BHI containing 0.05% DMSO was used as a control. After incubation at 37°C for 16 and 24 h, respectively, the absorbance at 630 nm (A_630_) was measured.

### Bacterial Cell Membrane Permeability

Fifty microliters of 1× PBS (pH 7.4) containing two-fold dilution of BT or melittin (positive control) at the indicated concentrations were added to a black 96-well plate (Corning no. 3904, Corning, NY, United States). Exponential-phase enterococcal cells were washed with 1× PBS, and their concentrations were adjusted to ∼1 × 10^8^ CFU/mL. Fluorescent dyes SYTOX, DiSC3(5), and PI were added to the bacterial suspensions to obtain final concentrations of 5, 2, and 10 μM, respectively, and incubated at room temperature in the dark for 30 min. Fifty microliters of the bacteria/fluorescent dye mixture were added to each well containing an antimicrobial agent, and the fluorescence intensity was measured every 5 min using a spectrophotometer (EnVision, PerkinElmer, United States) for 30–60 min with excitation/emission wavelengths of 485 nm/525 nm, 622 nm/670 nm, and 535 nm/617 nm for SYTOX, DiSC3(5), and PI, respectively (Kim et al., 2018; Porto et al., 2018; Ma et al., 2019).

### Scanning Electron Microscopy (SEM) and Transmission Electron Microscopy (TEM)

Enterococcal cultures in the mid-logarithmic growth phase were diluted in BHI broth to ∼1 × 10^8^ CFU/mL, cultured with 5 × MIC of BT with shaking at 180 rpm for 1 h, centrifuged, and washed with 1× PBS. As a control, the bacteria were exposed to BHI in 0.05% DMSO. The specimens were observed using SEM (Hitachi, Tokyo, Japan) and TEM (Hitachi, Tokyo, Japan).

### Checkerboard Assay

The antibiotic synergy test was performed as previously described (Mataraci and Dosler, 2012). Briefly, BT solutions were serially diluted two-fold and combined with conventional antibiotics that had been serially diluted two-fold in a 96-well plate and prepared in the presence of enterococcal cells at a final concentration of ∼1 × 10^6^ CFU/mL. After incubation for 16–20 h, the fractional inhibitory concentration (FIC) index was calculated as follows: FIC = MIC_A_ in combination/MIC_A_ alone + MIC_B_ in combination/MIC_B_ alone. FIC ≤ 0.5 indicates synergy, 0.5 < FIC ≤ 4 indicates no interaction, and FIC > 4 indicates antagonism.

### Biofilm Determination

To determine the optimal culture conditions for enterococcal biofilm formation, overnight cultures of enterococci were diluted with BHI culture medium in the presence or absence of serial concentrations of glucose (GLU) (BHI-g) to a final concentration of ∼1 × 10^6^ CFU/mL, at different incubation temperatures (25 and 37°C), and for different incubation periods (24 and 48 h) to detect the biofilm-forming ability of *E. faecalis* ATCC 29212 and *E. faecium* U101. Biofilm biomass was quantified using crystal violet (CV) staining. Briefly, planktonic cells were removed by washing with 1× PBS, and 100 μL of 0.25% CV (w/v) was added to each well, incubated at room temperature for 10 min, and washed with 1× PBS to remove unbound CV. The wells were air-dried, and A_570_ was measured (Mishra et al., 2015).

### Biofilm Inhibition and Eradication Assay

Overnight cultures of *E. faecalis* and *E. faecium* were diluted with BHI-g broth containing serially diluted BT to obtain a final concentration of ∼1 × 10^6^ CFU/mL for biofilm inhibition determination. After incubation at 37°C (for *E. faecalis*) or 25°C (for *E. faecium*) for 24 h, the planktonic cells were removed with 1× PBS, and the biofilms were stained with CV as described above.

Overnight cultured *E. faecalis* and *E. faecium* were diluted 1:200 with BHI-g broth and incubated at 37°C for *E. faecalis* or 25°C for *E. faecium* for 24 h to monitor biofilm eradication. Planktonic cells were removed by washing with 1× PBS, and the remaining biofilms were treated with serially diluted BT. After a 24 h incubation, planktonic cells were removed, and 200 μL of 2H-tetrazolium-5-carboxanilide (XTT, 0.2 μg/mL) containing phenazine methosulfate (PMS, 0.02 μg/mL) was added to each well. After incubation at 37°C for 2 h, A_490_ was detected as the metabolic activity of live cells in the biofilms (Nesse et al., 2015).

### Animal Models

The study design and animal experiments were approved by the Ethics Committee of the Third Xiangya Hospital of Central South University, Changsha, China (No. 2019sydw0211). Female, 6-week-old, outbred ICR mice (SJA Lab. Animal Co. Ltd. Changsha, China) with a mean weight of 25 g were used in this study. BT was dissolved in a Kolliphor/ethanol mixture (1:1, v/v) and diluted 1:10 with saline to the indicated concentrations before use. Enterococci were cultured overnight in BHI broth and washed with saline. Each mouse was intraperitoneally (i.p.) injected with 500 μL of 1 McFarland bacterial suspension containing 5% mucin. After 30 min of injection, antibiotics or BT were administered as previously reported (Kim et al., 2019): vehicle (5% Kolliphor + 5% ethanol, i.p.), BT (30 mg/kg, i.p.), and GEN or TOB (30 mg/kg subcutaneously [s.c.]). Antimicrobial agents were administered every 12 h for 3 days. The mice were euthanized 12 h after the last treatment, and their spleens and kidneys were excised and homogenized. The colonies were counted after 10-fold dilution on sheep blood agar.

### Resistance Induction by Sub-MIC

Resistance induction was performed as previously described (Friedman et al., 2006) with minor modifications. The MICs of BT and ciprofloxacin (CIP) against *E. faecalis* ATCC 29212 and *E. faecium* U101 were determined as described above. Then, 5 μL of bacterial suspension in the wells of 0.5 × MIC was 1,000-fold diluted with MH broth for further antimicrobial susceptibility testing as described above for the next-day antimicrobial susceptibility test. The protocol was followed for a 15-day period.

### One-Step Frequency of Resistance (FOR)

One-step frequency of resistance (FOR) was performed as previously described (Imai et al., 2019) with minor modifications. Briefly, *E. faecalis* ATCC 29212 and *E. faecium* U101 from exponential cultures were washed in 1× PBS and inoculated onto BHI plates (*n* = 10 plates/group) containing 1–16 × MIC of BT, at a density of 5 × 10^7^ CFU per plate. After incubation at 37°C for 48 h, the colonies on the plate were counted, and one-step FOR was calculated as: CFU (after incubation)/CFU (inoculated) × 100%.

### Statistical Analysis

All statistical analyses were performed and graphs were constructed using GraphPad Prism (v8.0). All assays were performed at least in triplicates. The data were analyzed using Student’s *t*-test or one-way ANOVA. A significance level of 0.05 was used for all statistical tests.

## Results

### BT Showed Strong Antimicrobial Activity Against *E. faecalis* and VAN-Resistant *E. faecium*

The MICs of BT against *E. faecalis* and *E. faecium* ranged from 1–4 μg/mL to 0.5–2 μg/mL, respectively. Unlike VAN (a bacteriostatic antibiotic for enterococci with MBC > 32 μg/mL), BT also showed effective bactericidal effects against enterococci with MBCs of 2–8 μg/mL. Furthermore, BT showed the same activity even against VAN-resistant *E. faecalis* and *E. faecium* (VRE) and other clinical isolates with different resistance patterns (Table 1 and Supplementary Table S1), which indicates that BT and VAN have different targets. DAP is a representative cyclic lipopeptide antibiotic used to treat gram-positive infections by disrupting bacterial cell membranes and inhibiting DNA, RNA, and protein synthesis (Heidary et al., 2018). Although BT has also been reported as a cell membrane-disrupting agent, it still shows high susceptibility to DAP-resistant strains (Table 1). In addition, since most of the *E. faecalis* strains are susceptible to β-lactams, AMP combined with GEN is the most recommended treatment (Thieme et al., 2018). However, in the present study, all clinical isolates of VRE remained resistant to AMP but were susceptible to BT (Table 1).

**TABLE 1:** Antimicrobial susceptibility test of BT and VAN against *enterococcus*.

**Strains**	**BT (μg/ml)**		**VAN (μg/ml)**		**DAP**		**AMP**	
	**MIC**	**MBC**	**MIC**	**MBC**	**MIC**	**MBC**	**MIC**	**MBC**
***E. faecalis***								
ATCC 29212	4	8	2	>32	4	8	1	4
EFS01	4	8	1	>32	4	8	1	2
EFS02	1	8	1	>32	4	8	1	1
EFS03	4	8	1	>32	4	8	1	4
EFS05	1	4	1	>32	4	16	1	2
EFS06	4	8	2	>32	4	16	1	4
EFS08	4	8	1	>32	4	8	1	4
EFS09	4	8	1	>32	4	8	1	4
EFS11	4	8	1	>32	4	16	1	32
EFSVRE1*	2	4	>32	>32	32	>32	>32	>32
EFSVRE2*	4	8	>32	>32	16	32	>32	>32
***E. faecium***								
EFM02	1	2	0.5	>32	4	16	2	8
EFM04	2	4	1	>32	16	32	>32	>32
EFM06	0.5	4	1	>32	8	16	2	8
EFM08	1	4	0.5	>32	16	16	>32	>32
EFM09	1	4	0.5	>32	8	8	>32	>32
EFM10	1	4	1	>32	8	16	4	16
EFM12	2	4	0.5	>32	4	8	>32	>32
EFM13	1	4	0.5	>32	16	32	>32	>32
EFM14	1	4	0.5	>32	8	8	1	4
EFM16	1	4	1	>32	8	16	>32	>32
EFM17	2	4	1	>32	4	16	>32	>32
U101*	1	2	>32	>32	16	>32	>32	>32

*VAN-resistant strains.

**FIGURE 1:**
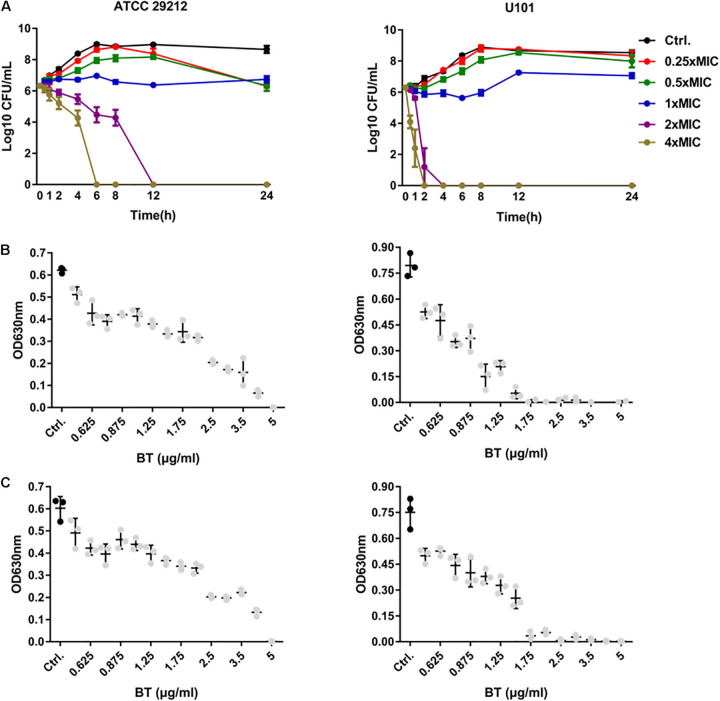
Antimicrobial effects of BT against *E. faecalis* and *E. faecium* planktonic cells. **(A)** Time-dependent antimicrobial effects of BT against ATCC 29212 and U101. Dose-dependent antimicrobial effects of BT against ATCC 29212 and U101 for **(B)** 16 h and **(C)** 24 h, respectively. Gray dots indicate *p* < 0.05, compared with the control group.

The bactericidal activity of BT was time-dependent and dose-dependent. As shown in Figure 1A, 2 × MIC of BT completely killed *E. faecalis* ATCC 29212 and *E. faecium* U101 within 12 and 4 h, respectively. Only 6 and 2 h were needed to eliminate the live cells of *E. faecalis* and *E. faecium*, respectively, in the presence of 4 × MIC of BT. Furthermore, by detecting the bacterial growth turbidity at 630 nm, there was an obvious dose-dependent growth inhibitory effect of sub-MIC of BT against enterococcal cells for either 16 h (Figure 1B) or 24 h treatment (Figure 1C). In addition, enterococci showed a high FOR by sub-MIC of CIP induction within 15 days; however, BT showed an extremely low FOR in enterococci either by serial passage experiments in the presence of sub-MIC of BT (Supplementary Figure S5A) or one-step FOR assay in the presence of 1–16 × MIC (Supplementary Figure S5B).

### BT Disrupted Bacterial Cell Membrane Integrity of *E. faecalis* and *E. faecium*

**FIGURE 2:**
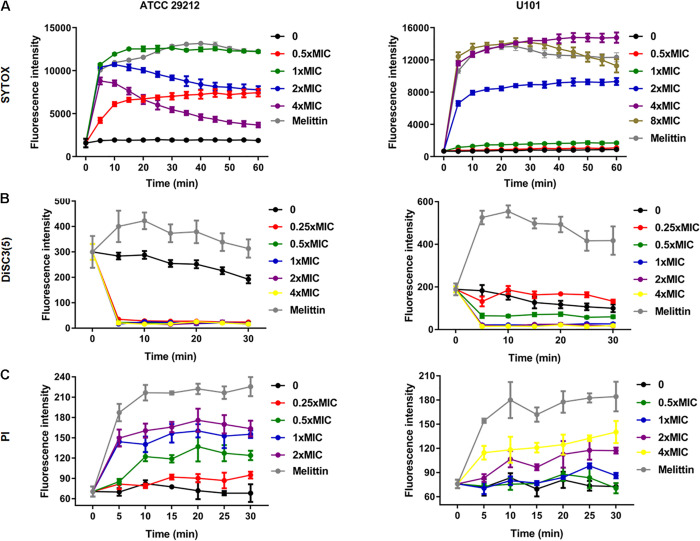
Bacterial cell membrane-disrupting effects of BT against Enterococcus. Bacterial cells were washed with PBS and treated with BT, and fluorescent dyes were used to monitor the disruption of cell membrane integrity. **(A)** SYTOX was used to stain the disrupted cell membranes, **(B)** DiSC3(5) was used to detect the exchange of intracellular potassium ions, and **(C)** PI bound to the DNA inside the cells with the disrupted cell membranes.

SYTOX Green, DiSC3(5), and PI are sensitive to changes in cell membrane integrity. In the present study, the antimicrobial peptide melittin was used as a positive control as it kills bacterial cells by disrupting their cell membranes and causing cell membrane depolarization (Jamasbi et al., 2016). Treatment of *E. faecalis* and *E. faecium* with 0.5–8 × MIC of BT considerably increased the fluorescence intensity of SYTOX Green (Figure 2A) and PI (Figure 2C) in a dose-dependent manner. However, unlike melittin, treatment with BT decreased the fluorescence intensity of DiSC3(5), indicating cell membrane hyperpolarization (Figure 2B).

**FIGURE 3:**
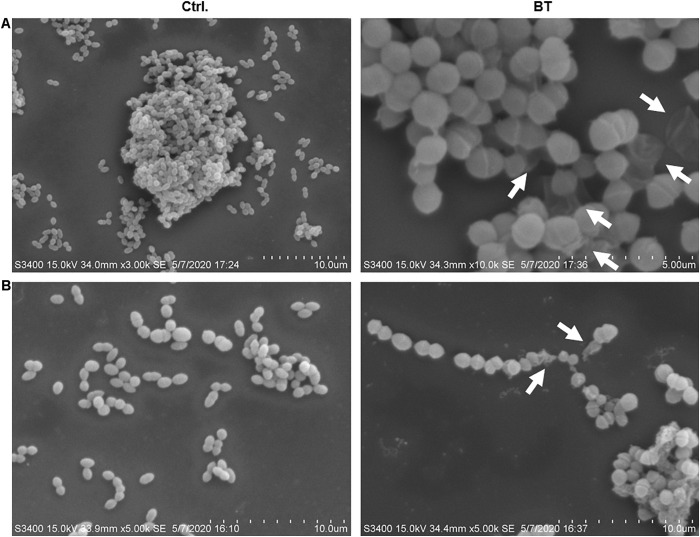
SEM images of BT treatment of **(A)** *Enterococcus faecalis* ATCC 29212 and **(B)** VAN-resistant *E. faecium* U101; 5 × MIC of BT was used for 1 h treatment, followed by fixation with 2% glutaraldehyde. The arrows indicate cell membrane interruption.

**FIGURE 4:**
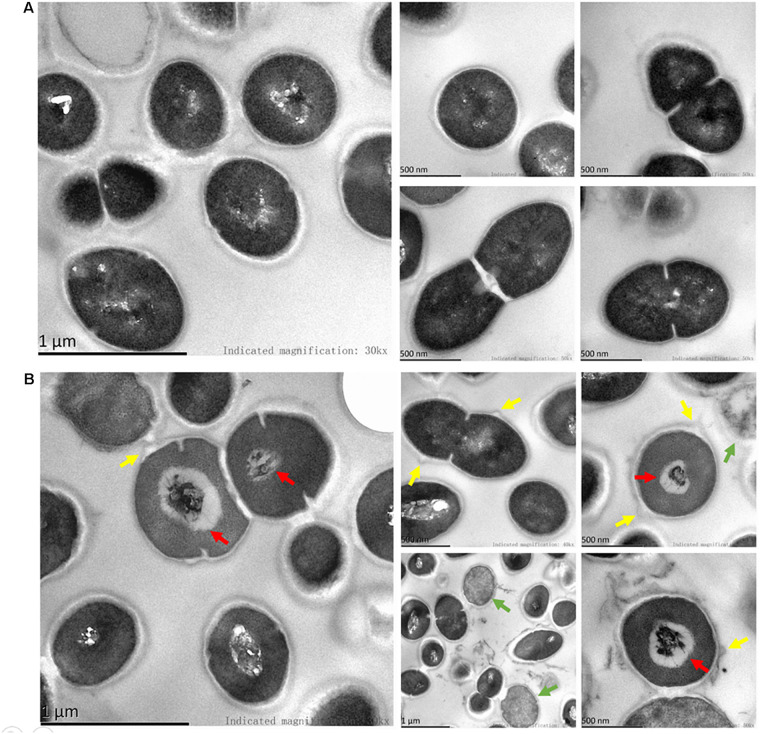
TEM images of BT treatment of *E. faecalis*. **(A)** Control group, the cells were treated with 0.05% DMSO. **(B)** The bacterial cells were treated with 5 × MIC of BT for 1 h. Green arrows indicate dead cells; red arrows indicate dense deposits; yellow arrows indicate cell surface blebbing.

SEM images revealed considerable disruption of the bacterial cell membranes of *E. faecalis* and *E. faecium* after 1 h of BT treatment. The surfaces of the bacterial cells were wrinkled and transparent, leading to cell lysis and necrosis (Figure 3). Similarly, TEM analysis revealed cell surface blebbing and dense deposits in BT-treated bacteria, indicating that there may be other mechanisms underlying the antimicrobial effects of BT in addition to cell membrane disruption (Figure 4).

### Synergistic Antimicrobial Effects Between BT and Conventional Antibiotics

**FIGURE 5:**
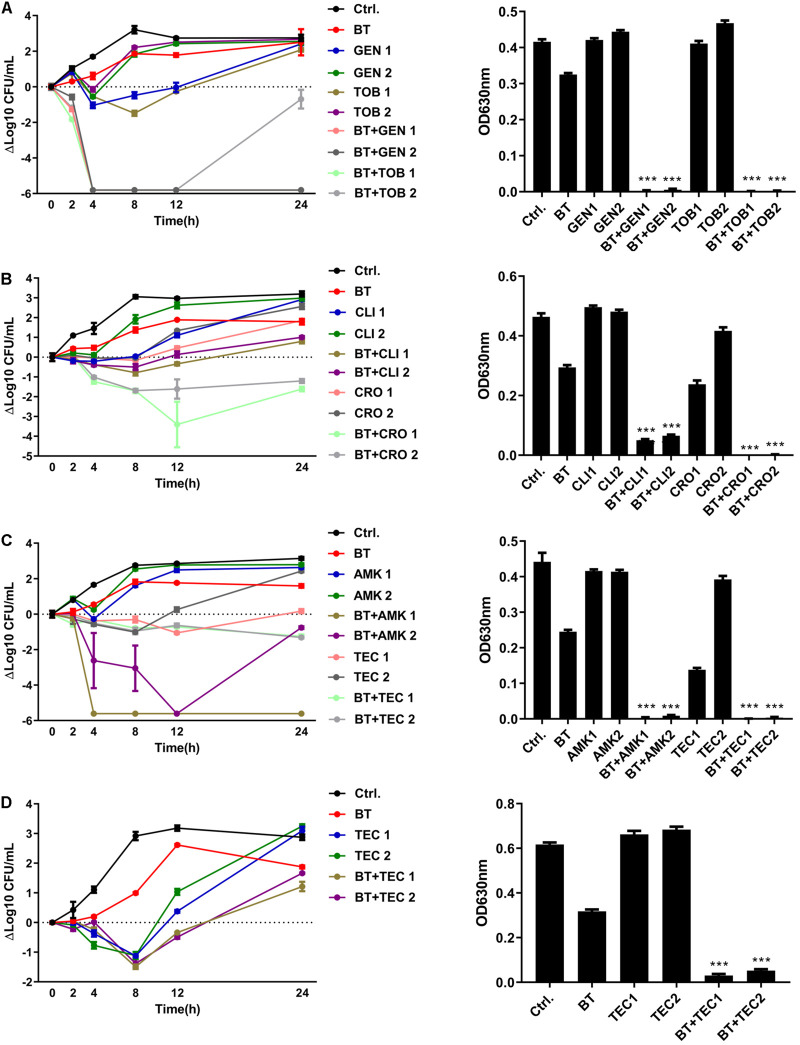
BT enhanced antimicrobial effects of antibiotics against *Enterococcus*. **(A)** BT + GEN/TOB or **(B)** BT + CLI/CRO or **(C)** BT + AMK/TEC against *E. faecalis* ATCC 29212; **(D)** BT + TEC against *E. faecium* U101. BT: 1 μg/mL for *E. faecalis* and 0.5 μg/mL for *E. faecium*, GEN1: 8 μg/mL, GEN2: 4 μg/mL, TOB1: 8 μg/mL, TOB2: 4 μg/mL, CLI1: 2 μg/mL, CLI2: 1 μg/mL, CRO1: 8 μg/mL; CRO2: 4 μg/mL, AMK1: 32 μg/mL, AMK2: 16 μg/mL, TEC1: 0.25 μg/mL, TEC2: 0.125 μg/mL. Left panel: Time-kill curves of the combinations of BT plus antibiotics. *Y*-axis represents the Δlog10 CFU/mL at each time point. Dotted line indicates the baseline of Δlog10 = 0. Right: Turbidity of the culture suspension (A_630_) at the time point of 24 h.

Bithionol disrupted the cell membrane integrity of *E. faecalis* and *E. faecium*, thus increasing the permeability of other antibiotics. A checkerboard method was used to determine the interactions between BT and other antibiotics. Many antibiotics, including AMK, CLI, CRO, GEN, TOB, and TEC, showed synergistic antimicrobial effects against *E. faecalis* ATCC 29212 with FIC ≤ 0.5. However, fewer antibiotics showed synergy with BT against *E. faecium* U101; only TEC showed synergistic effects with BT with FIC = 0.5 (Table 2 and Supplementary Figure S1). In addition, there was no synergistic effect between BT and DAP/AMP (Table 2). The sub-MICs of BT and the test antibiotics were then selected to evaluate the combined antimicrobial activity using time-kill assays (Figure 5). Neither the sub-MIC of BT at 1 μg/mL nor the sub-MICs of GEN/TOB, CLI/CRO, or AMK/TEC showed obvious bacterial growth inhibitory effects against *E. faecalis*; however, there was marked synergism in their antimicrobial effects when combined. For example, there was a 5.80 (Δlog10 CFU/mL) reduction in the BT + GEN (4 or 8 μg/mL) and BT + TOB (4 or 8 μg/mL) after 4 h treatment (Figure 5A); BT + CLI (1 μg/mL), BT + CLI (2 μg/mL), BT + CRO (4 μg/mL), and BT + CRO (8 μg/mL) reduced the number of live cells by Δlog10 CFU/mL of 0.50, 0.78, 1.69, and 1.70, respectively, after 8 h of treatment (Figure 5B); BT + AMK (16 μg/mL), BT + AMK (32 μg/mL), BT + TEC (0.125 μg/mL), and BT + TEC (0.25 μg/mL), could reduce the number of live cells by Δlog10 CFU/mL of 3.05, 5.61, 0.95, and 0.79, respectively, at the time point of 8 h (Figure 5C). Although the bacterial counts increased to some extent over 24 h, probably due to the consumption of BT or resistant cell emergence over time, the combinations of antimicrobial agents still showed more significant growth inhibitory effects than the monotherapies (Figure 5, right panel). As *E. faecium* was more susceptible to BT than *E. faecalis*, *E. faecium* U101 was treated with 0.5 μg/mL BT combined with the sub-MIC of TEC. The sub-MIC of TEC significantly enhanced the antimicrobial activity of BT within 8 h. After 8 h treatment, BT + TEC (0.125 and 0.25 μg/mL) could significantly reduce the live cells by Δlog10 CFU/mL of 1.38 and 1.49, respectively (Figure 5D). However, the synergistic activities between BT and the other antibiotics were strain-dependent (Table 3). BT and the antibiotics showed varied effects against the clinical isolates, probably due to varied genetic backgrounds and resistance patterns, which indicates that *in vitro* combination-related assays must be performed before the use of such combinations in clinical settings.

**TABLE 2:** FIC of antimicrobial combination against *E. faecalis* and *E. faecium.*

**Antibiotics**	***E. faecalis***			**Outcome**	***E. faecium***			**Outcome**
	**Exp1**	**Exp2**	**Exp3**		**Exp1**	**Exp2**	**Exp3**	
AMK	0.38	0.38	0.38	Synergy	0.5	0.5	0.75	No interaction
CLI	0.31	0.19	0.38	Synergy	0.75	0.5	0.75	No interaction
CRO	0.25	0.25	0.31	Synergy	0.63	0.5	0.75	No interaction
GEN	0.38	0.28	0.31	Synergy	0.56	0.75	1	No interaction
TOB	0.38	0.31	0.31	Synergy	0.5	0.5	0.75	No interaction
TEC	0.5	0.5	0.5	Synergy	0.5	0.5	0.5	Synergy
DAP	0.5	0.75	0.75	No interaction	0.75	1	1	No interaction
AMP	0.56	0.56	0.56	No interaction	1	2	1	No interaction

**E. faecalis*: ATCC29212; *E. faecium*: U101. The experiments were repeated at least duplicate, and the optimal values of optimal FIC were shown.*

**TABLE 3:** The optimal FIC between BT and antibiotics against *E. faecalis* and *E. faecium*.

**Antibiotics**	***E. faecalis***			***E. faecium***		
	**EF02**	**EF05**	**EF11**	**EFM04**	**EFM08**	**EFM12**
AMK	0.31	0.31	0.25	0.75	0.75	0.38
CLI	0.63	0.5	0.5	1	2	0.5
CRO	0.5	0.38	0.31	2	2	0.53
GEN	0.28	0.31	0.5	1.5	1	0.5
TOB	0.38	0.31	0.31	2	1	1
TEC	0.75	0.75	0.5	0.75	2	1

**E. faecalis*: ATCC29212; *E. faecium*: EFM09. The experiments were repeated at least duplicate, and the optimal values of optimal FIC were shown.*

### Antibiofilm Effects of BT

**FIGURE 6:**
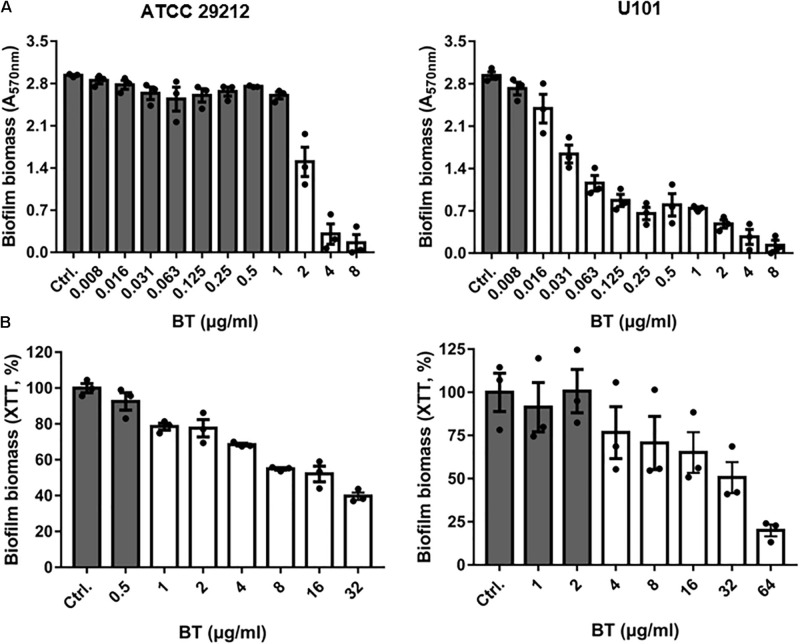
Antibiofilm effects of BT against *Enterococcus*. **(A)** Dose-dependent biofilm inhibition of BT using crystal violet staining. **(B)** Dose-dependent biofilm eradication of BT using XTT staining. The white column indicates *p* < 0.05 compared with the control group.

Although the biofilm-forming ability of enterococci has been widely reported (Ch’ng et al., 2019), the optimal culture conditions for *E. faecalis* and *E. faecium* biofilm formation are unclear. In the present study, we found that the optimal culture conditions for *E. faecalis* ATCC 29212 biofilm formation comprised BHI broth with 2% GLU incubated at 37°C for 24 h. BHI broth with 2% GLU incubated at 25°C for 24 h was the optimal biofilm formation condition for *E. faecium* U101 (Supplementary Figure S2). BT significantly inhibited biofilm formation by ATCC 29212 and U101 at 2 and 0.016 μg/mL, respectively, in a dose-dependent manner (Figure 6A). Similarly, BT showed obvious biofilm inhibitory effects against enterococcal clinical isolates with half maximal inhibitory concentration (IC_50_) values of 0.17–1.49 μg/mL (Supplementary Figures S3A,B). BT eradicated 24 h-preformed biofilms at concentrations of 1 and 4 μg/mL for ATCC 29212 and U101, respectively (Figure 6B). Biofilms formed by the clinical isolates also could be removed by BT at concentrations of 0.25–8 μg/mL (Supplementary Figures S3C,D).

### Antimicrobial Efficacy in a Murine Peritonitis Model

**FIGURE 7:**
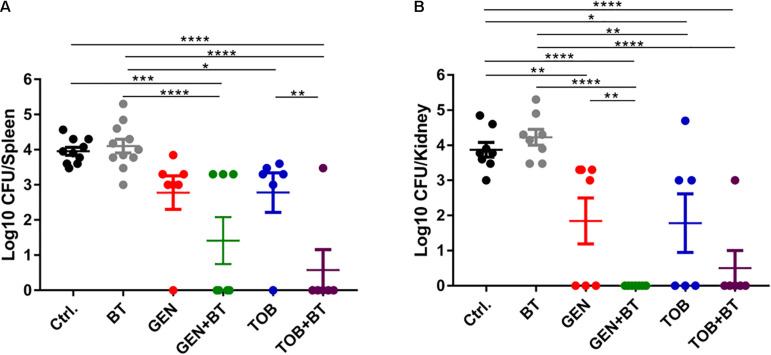
Bacterial quantities displayed as log10 (CFU/mice) in all treatment groups. **(A)** Spleen and **(B)** Kidney. After 1 h following i.p. injection of 500 μL of 1 McF of *E. faecalis* ATCC 29212 with 5% mucin, BT 30 mg/kg (i.p.) combined with GEN/TOB 30 mg/kg (s.c.) was administered twice daily for a total of 3 days. *p < 0.05, **p < 0.01, ***p < 0.001, ****p < 0.0001. *N* = 6–11 mice per group.

By i.p. infection with 500 μL of 1–4 McFarland *E. faecalis* ATCC 29212, relatively high bacterial loads were found in the spleen, kidney, and liver of mice (Supplementary Figure S4A). To minimize the ill effects of infection, 1 McF bacterial concentration was selected for subsequent experiments. However, there was no or very limited bacterial load in these organs even when the mice were infected with a high density of *E. faecium* U101, which was probably due to the lower virulence of *E. faecium* than that of *E. faecalis* (Supplementary Figure S4B). In the present study, monotherapy with BT or GEN/TOB showed none or very limited antimicrobial effects, respectively, in the *E. faecalis*-related peritonitis model. However, EP plus GEN/TOB combination treatment was significantly more effective than the control group and the constituent monotherapy groups, causing a significant decrease in the bacterial loads in the spleen and kidney (Figure 7).

## Discussion

In this study, BT, an antiparasitic drug, exhibited effective antimicrobial and antibiofilm activity against *E. faecalis* and *E. faecium in vitro* without inducing antimicrobial resistance. In combination with GEN/TOB, BT significantly reduced the bacterial load in different organs in a murine peritonitis model *in vivo*.

Bithionol showed effective antimicrobial activity against enterococci *in vitro* by disrupting the bacterial cell membrane. Barr et al. (1965) first detected the antimicrobial activity of BT and Kim et al. (2019) reported that BT could effectively kill MRSA persister cells with MICs of 0.5–2 μg/mL. Using molecular dynamics and biomembrane-mimicking giant unilamellar vesicle assays, Kim et al., 2019 found that BT could penetrate and embed in MRSA bacterial-mimicking lipid bilayers and increase membrane fluidity. In this study, we confirmed that BT exhibited significant antimicrobial effects against *E. faecium* (including VAN-resistant strains) with MICs of 0.5–2 μg/mL. However, BT showed weaker antimicrobial effects against *E. faecalis* than against *E. faecium*, as evidenced by MICs of 1–4 μg/mL. By staining with SYTOX, DiSC(3)5, and PI, we further confirmed that the underlying mechanism of the anti-enterococcal effect of BT involved the disruption of the bacterial cell membrane. Interestingly, BT could also effectively kill *E. faecalis* and *E. faecium* clinical strains that are resistant to DAP (an Food and Drug Administration (FDA)-approved antibiotic that targets the bacterial cell membrane) (Table 1). Thus, we speculate that there might be another underlying mechanism after BT disrupts the cell membrane and intrudes into the bacterial cells.

Biofilms are known to be far more resistant than their planktonic counterparts (Venkatesan et al., 2015). Enterococcal strains form biofilms on wounds and medical apparatus and cause catheter-related infections that are refractory to treatment (Ch’ng et al., 2019). The most harmful component of biofilms is the inner persister cells, a dormant cell that is metabolically active, but without any proliferative activity, and is extremely resistant to antibiotics (Yan and Bassler, 2019). In the present study, BT was found to be efficacious in inhibiting biofilm formation and eradicating preformed biofilms of *E. faecalis* and *E. faecium*. BT can completely kill stationary-phase MRSA and its biofilm persisters within 24 h (Kim et al., 2019). The persistence killing efficacy of BT was probably also the main reason for its antibiofilm efficacy against enterococci.

One of the worst shortcomings of drug repurposing for antibiotic development is its side effects (Thangamani et al., 2015). Four to 6 weeks of AMP combined with GEN treatment is the first choice to treat infective endocarditis caused by *E. faecalis*. However, the emergence of high-level GEN resistance and severe side effects (such as ototoxicity and nephrotoxicity) caused by GEN cannot be ignored (Thieme et al., 2018). Thus, a substitute for AMP with enhanced antimicrobial effects and lower resistance is urgently needed when combined with conventional antibiotics against *E. faecalis*-related infections. In the present study, besides aminoglycosides (GEN and AMK), BT could have a synergistic effect with a wide range of conventional antibiotics such as CRO and TEC (Figure 5), which might result in better efficacy but fewer side effects than combined with aminoglycosides. In addition, BT selectively binds to bacterial cell membranes without affecting mammalian cell membranes at concentrations up to 64 μg/mL (Kim et al., 2019). BT was safe even at a high dose of 240 mg/kg for more than 30 days of treatment in a mouse study, investigating *in vivo* toxicity (Ayyagari et al., 2016; Kim et al., 2019). Thus, the high therapeutic index and effective antimicrobial activity of BT make it an antibiotic with great potential for clinical use.

The resistance to BT has an extremely low probability. Similar to DAP, the phospholipid bilayer of the cell membrane is the major target of BT (Kim et al., 2019). Resistance to DAP has been reported in the LiaFSR system, and cardiolipin synthase and glycerophosphoryl diester phosphodiesterase induced changes in cell surface charge, redistribution of cardiolipin, and in blocking membrane association and oligomerization (Miller et al., 2016). However, BT still showed high susceptibility even against DAP-resistant strains in the present study. In addition, there was no resistance to either the sub-MIC BT-inducing assay or one-step FOR tests. Thus, the structure of BT is probably better than DAP because of its better antimicrobial activity and lower resistance-inducing probability.

Bithionol showed synergy with many antibiotics, probably owing to its ability to disrupt bacterial cell membranes, thereby facilitating the penetration of other antibiotics into the cells. Although BT enhanced the antimicrobial effects of other antibiotics *in vitro*, monotherapy with BT had no effect on bacterial elimination in the thigh infection mouse model (Kim et al., 2019) and a peritonitis mouse model (Figure 7). Furthermore, there was no significant difference in the bacterial loads between the control and BT/BT + antibiotic-treated livers (data not shown). This could probably be due to the unsatisfactory *in vivo* pharmacokinetic profile of BT. Our results demonstrate the need for structural and pharmacokinetic optimization of BT to enhance its antimicrobial efficacy and minimize its *in vivo* toxicity before BT is used in clinical settings.

## Conclusion

Bithionol, an anthelminthic drug, is an effective bactericidal agent against both planktonic cells and biofilms of enterococci by disrupting their cell membrane. BT could also be synergistic with conventional antibiotics against *E. faecalis in vitro* and *in vivo*. The effective antimicrobial efficacy and low toxicity make BT a valuable alternative candidate for treating VRE-related infections.

## Data Availability Statement

The original contributions presented in the study are included in the article/Supplementary Material, further inquiries can be directed to the corresponding author/s.

## Ethics Statement

The animal study was reviewed and approved by The Animal Ethics Committee of Third Xiangya Hospital.

## Author Contributions

PS, YW (2nd author), and YW (9th author) designed and performed the experiments and wrote the manuscript. YiL, LZ, and SL performed the experiments and data collection. XZ, YaL, and LX performed the experiments and revised the manuscript. All authors read and approved the final manuscript.

## Conflict of Interest

The authors declare that the research was conducted in the absence of any commercial or financial relationships that could be construed as a potential conflict of interest.

## Supplementary Material

Supplementary Figure 1 Efficacy of antimicrobial combinations of BT and antibiotics against **(A)** *E. faecalis* ATCC 29212 and **(B)** VAN-resistant *E. faecalis* U101 using the chessboard dilution assay.

Supplementary Figure 2 Effects of culture medium, temperature, glucose addition, and incubation time on biofilm formation of *E. faecalis* ATCC 29212 and *E. faecium* U101. **(A)** 24 h incubation; **(B)** 48 h incubation. Red arrows indicate the wells with the strongest biofilm formation. The biofilms were quantified using crystal violet staining.

Supplementary Figure 3 Antibiofilm effects of BT against clinical isolates. **(A)** *E. faecalis* and **(B)** *E. faecium* clinical strains by crystal violet staining. Biofilm eradication effects of BT against **(C)** *E. faecalis* and **(D)** *E. faecium* clinical strains by XTT staining. The white column indicates *p* < 0.05 compared with the control group.

Supplementary Figure 4 Bacterial loads in spleen, kidney, and liver after 3 days of infection by **(A)** *E. faecalis* ATCC 29212 and **(B)** VAN-resistant *E. faecium* U101. *Enterococcus* was infected by i.p. injection with 500 μL of the bacterial suspension at the indicated concentrations (1–4 McF) with 5% mucin. *N* = 3 mice per group.

Supplementary Figure 5 Antimicrobial resistance induction by BT. **(A)** Fold changes of MIC in *E. faecalis* ATCC 29212 and *E. faecium* U101 in the presence of sub-MIC of BT and CIP by serial-passage assay for a total of 15 passages. The initial MICs of CIP against ATCC 29212 and U101 were 0.5 and 2 μg/mL, respectively. P indicates a parallel experiment. **(B)** Frequencies of resistance of *E. faecalis* ATCC 29212 and *E. faecium* U101 to BT at 1 × MIC to 16 × MIC. The experiments were repeated five times on different days with no resistance. A representative image is shown.
