# Supplementary figures and images for: Drug Repurposing: In vitro and in vivo Antimicrobial and Antibiofilm Effects of Bithionol Against Enterococcus faecalis and Enterococcus faecium
Source: Front Microbiol. 2021 May 6;12:579806. doi: 10.3389/fmicb.2021.579806 (PMC8138570; doi:10.3389/fmicb.2021.579806)

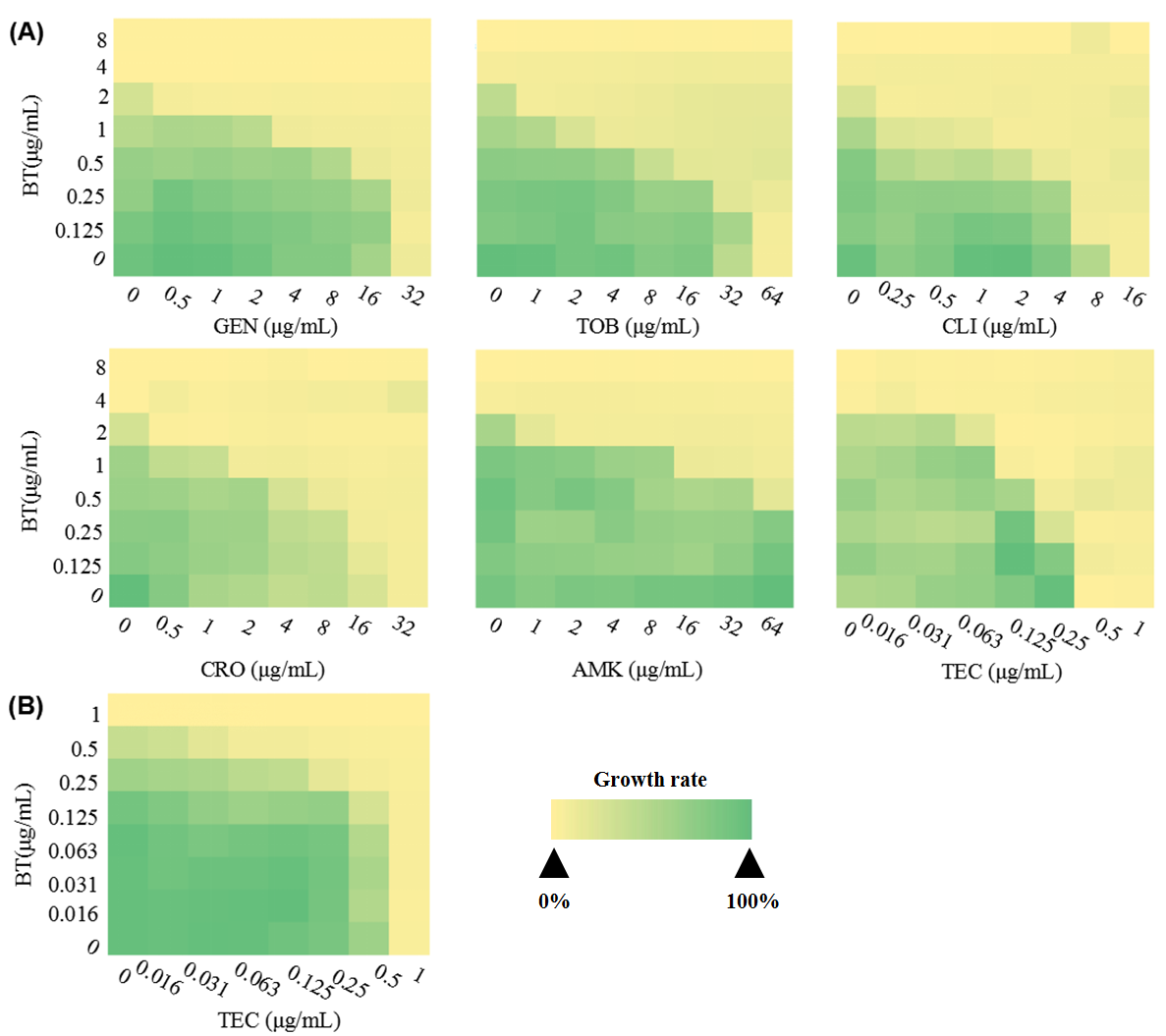

Supplement: Supplementary Figure 1 — Efficacy of antimicrobial combinations of BT and antibiotics against (A) E. faecalis ATCC 29212 and (B) VAN-resistant E. faecalis U101 using the chessboard dilution assay. [file Image_1.tif]

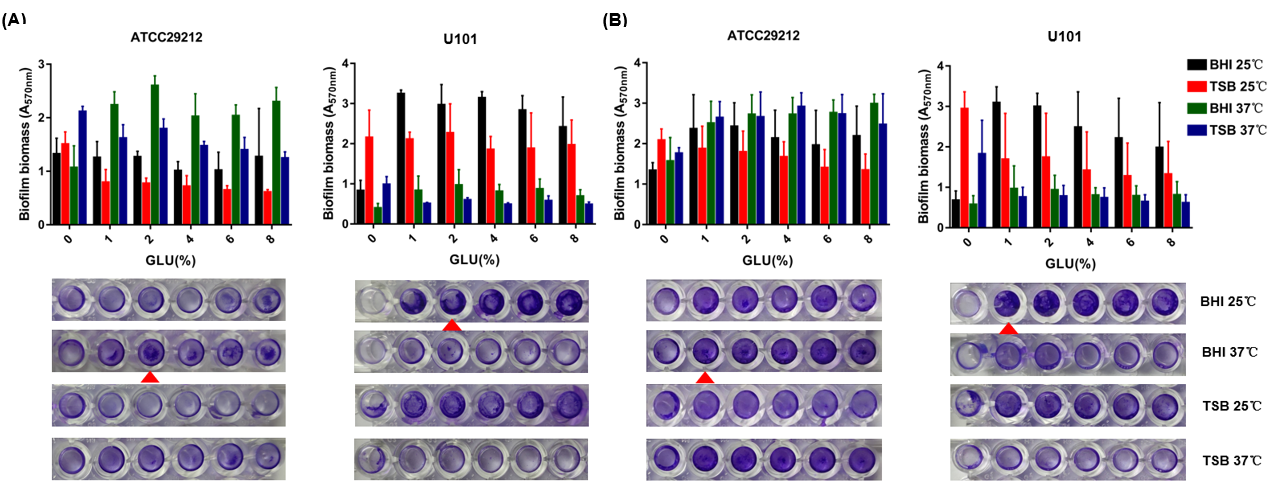

Supplement: Supplementary Figure 2 — Effects of culture medium, temperature, glucose addition, and incubation time on biofilm formation of E. faecalis ATCC 29212 and E. faecium U101. (A) 24 h incubation; (B) 48 h incubation. Red arrows indicate the wells with the strongest biofilm formation. The biofilms were quantified using crystal violet staining. [file Image_2.TIF]

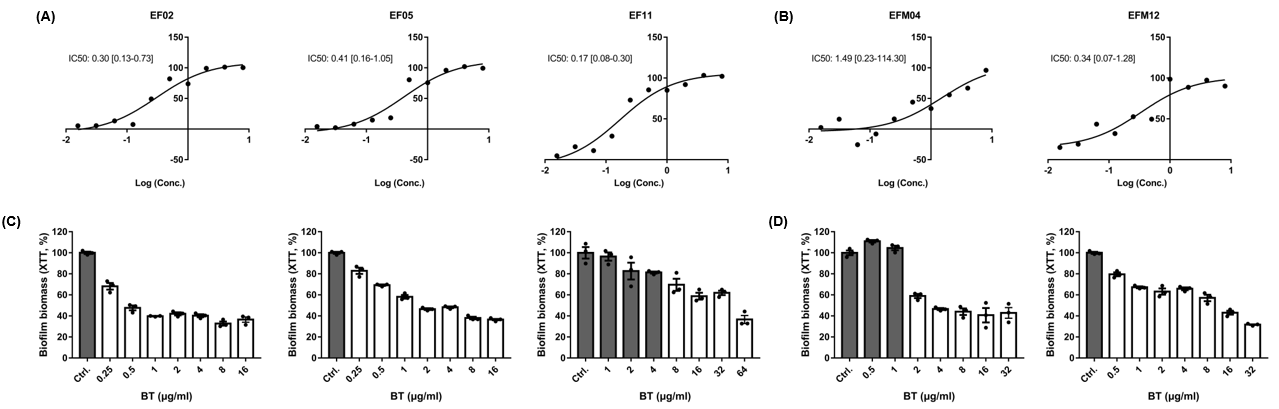

Supplement: Supplementary Figure 3 — Antibiofilm effects of BT against clinical isolates. (A) E. faecalis and (B) E. faecium clinical strains by crystal violet staining. Biofilm eradication effects of BT against (C) E. faecalis and (D) E. faecium clinical strains by XTT staining. The white column indicates p < 0.05 compared with the control group. [file Image_3.TIF]

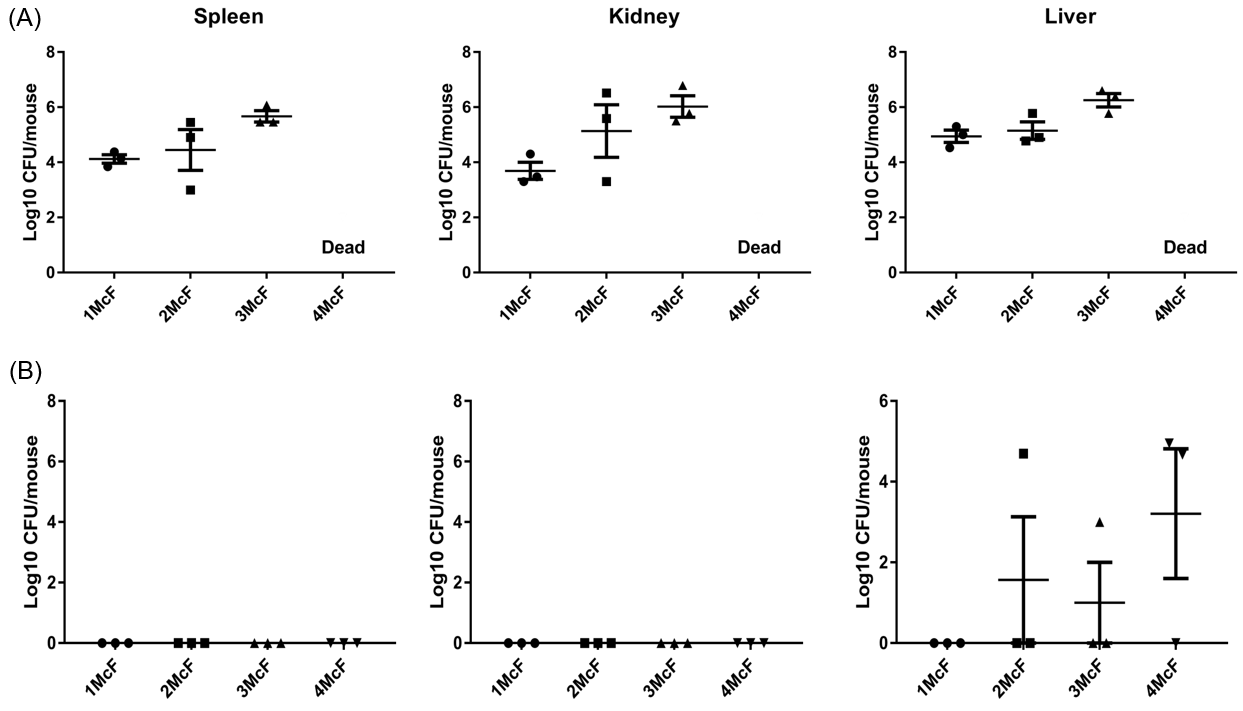

Supplement: Supplementary Figure 4 — Bacterial loads in spleen, kidney, and liver after 3 days of infection by (A) E. faecalis ATCC 29212 and (B) VAN-resistant E. faecium U101. Enterococcus was infected by i.p. injection with 500 μL of the bacterial suspension at the indicated concentrations (1–4 McF) with 5% mucin. N = 3 mice per group. [file Image_4.TIF]

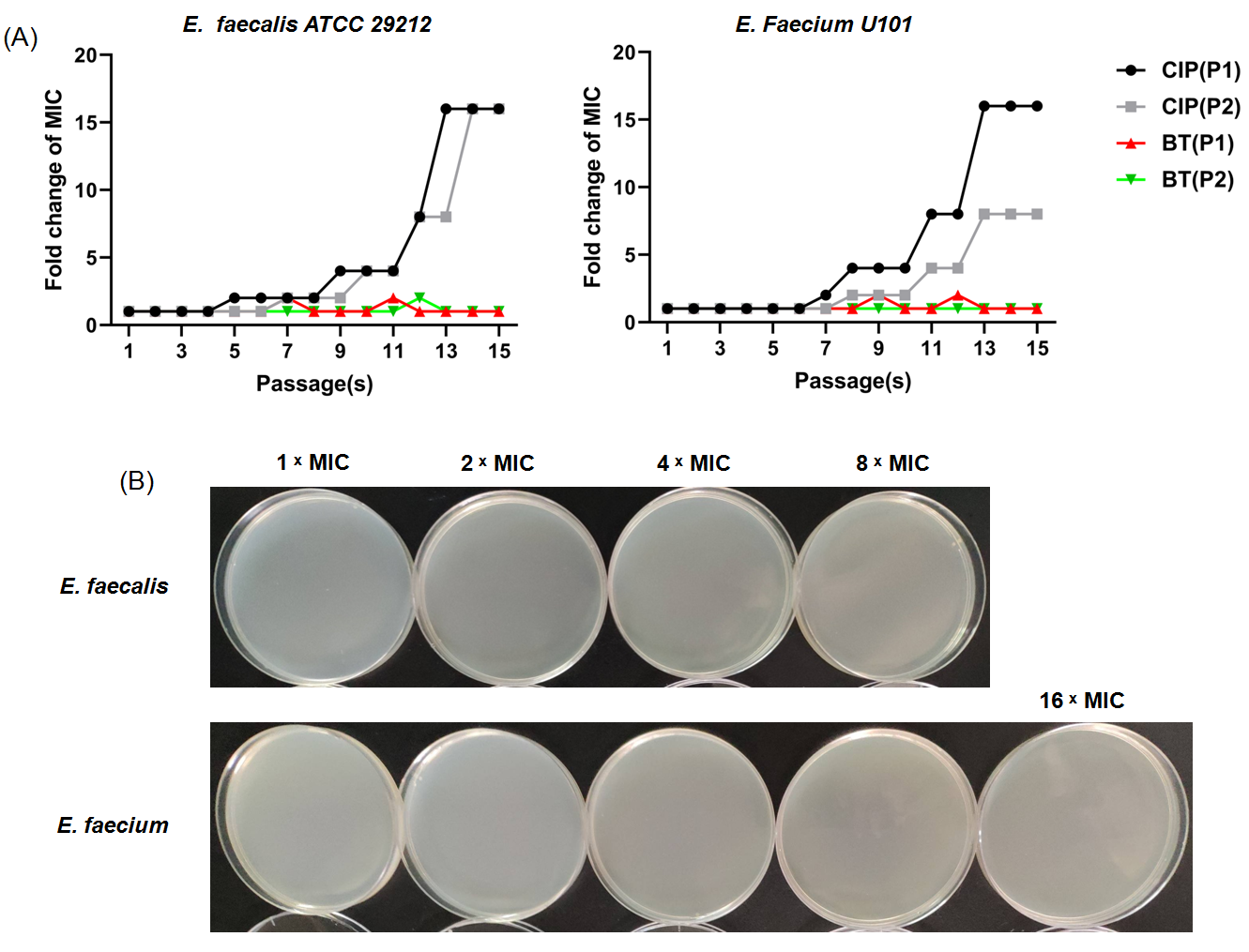

Supplement: Supplementary Figure 5 — Antimicrobial resistance induction by BT. (A) Fold changes of MIC in E. faecalis ATCC 29212 and E. faecium U101 in the presence of sub-MIC of BT and CIP by serial-passage assay for a total of 15 passages. The initial MICs of CIP against ATCC 29212 and U101 were 0.5 and 2 μg/mL, respectively. P indicates a parallel experiment. (B) Frequencies of resistance of E. faecalis ATCC 29212 and E. faecium U101 to BT at 1 × MIC to 16 × MIC. The experiments were repeated five times on different days with no resistance. A representative image is shown. [file Image_5.TIF]
