# Supplementary material for: Drug Repurposing: In vitro and in vivo Antimicrobial and Antibiofilm Effects of Bithionol Against Enterococcus faecalis and Enterococcus faecium
Source: Front Microbiol. 2021 May 6;12:579806. doi: 10.3389/fmicb.2021.579806 (PMC8138570; doi:10.3389/fmicb.2021.579806)
Supplement: Supplementary file 6 [file Table_1.docx]

Table S1. Antimicrobial susceptibility test of BT and VAN against *enterococcus*

| **Strains** | Source | Antibiotic resistance | | | | | | | |  |
| --- | --- | --- | --- | --- | --- | --- | --- | --- | --- | --- |
|  |  | E | LEV | P | TET | MOX | GEN^hi^ | LZD | TEG | |
| ***E. faecalis*** |  |  |  |  |  |  |  |  |  | |
| ATCC 29212 | - | I | S | S | S | S | S | S | S | |
| EFS01 | U | R | R | R | R | R | R | S | S | |
| EFS02 | B | I | R | S | S | R | S | S | S | |
| EFS03 | U | R | R | R | R | R | R | S | S | |
| EFS05 | U | S | I | S | R | S | S | S | S | |
| EFS06 | U | R | R | S | R | R | R | S | S | |
| EFS08 | B | R | R | R | R | S | R | S | S | |
| EFS09 | PE | I | S | S | S | S | S | S | S | |
| EFS11 | U | R | R | S | R | R | R | S | S | |
| EFSVRE1* | U | I | R | R | R | R | R | S | S | |
| EFSVRE2* | U | R | R | R | R | R | R | S | S | |
| ***E. faecium*** |  |  |  |  |  |  |  |  |  | |
| EFM02 | U | R | R | R | S | R | S | S | S | |
| EFM04 | U | R | R | R | S | R | R | S | S | |
| EFM06 | U | R | R | R | S | R | R | S | S | |
| EFM08 | U | R | R | R | S | R | R | S | S | |
| EFM09 | U | R | R | R | R | R | S | S | S | |
| EFM10 | PE | R | R | R | S | R | S | S | S | |
| EFM12 | U | R | R | R | S | R | S | S | S | |
| EFM13 | U | R | R | R | R | R | R | S | S | |
| EFM14 | PE | R | R | R | R | R | R | S | S | |
| EFM16 | U | R | R | R | S | R | R | S | S | |
| EFM17 | U | R | R | R | R | R | R | S | S | |
| U101* | U | R | R | R | R | R | R | S | S | |

E: erythromycin; LEV: levofloxacin; P: penicillin G; TET: tetracycline; MOX: moxifloxacin; GEN^hi^: high concentration of gentamycin; LZD: linezolid; TEG: tigecycline; U: urine; B: Blood; PE: pleural effusion; R: resistant; I: intermediate; S: susceptible.
